# Long noncoding RNAs as regulators of cancer immunity

**DOI:** 10.1002/1878-0261.12413

**Published:** 2018-12-13

**Authors:** Nerina Denaro, Marco Carlo Merlano, Cristiana Lo Nigro

**Affiliations:** ^1^ Oncology Department S. Croce & Carle Teaching Hospital Cuneo Italy; ^2^ Laboratory of Clinical Trials Laboratory Department S. Croce & Carle Teaching Hospital Cuneo Italy

**Keywords:** long noncoding RNAs, lncRNAs, cancer immunity

## Abstract

Long noncoding RNAs (lncRNAs) are increasingly known to be important in cancer as they directly interact with the cell cycle, proliferation pathways and microbiome balance. Moreover, lncRNAs regulate the immune system: they do not directly encode proteins of innate or adaptive immunity, but regulate immune cell differentiation and function, such as dendritic cell activity, T cell ratio and metabolism. The result of this complex interaction is that lncRNAs regulate cancer processes through a complex multimodal system involving immunity, metabolism and infection. The possible functions of lncRNAs and their roles in the regulation of cancer immunity will be reported and discussed in the present review. Recent studies showed their function as regulators in the tumour microenvironment (TME), epithelial–mesenchymal transition, microbiota, metabolism and immune cell differentiation. However, there is not much knowledge regarding their roles in cancer immunity regulation. Thus, the main aim of this review is to describe lncRNAs that have specifically been associated with immunity, the immune cycle and the TME.

**Abbreviations**
ACOD1: aconitate decarboxylase 1
ANRIL: antisense noncoding RNA in the INK4 locus
APC/DCs: antigen‐presenting cells/dendritic cells
BLACAT1: bladder cancer‐associated transcript 1
C/EBPβ: CCAAT‐enhancer‐binding proteins
CHOP: C/EBP homologous protein
COX2: cyclooxygenase‐2
CRNDE: colorectal neoplasia differentially expressed
DCs: dendritic cells
EMT: epithelial–mesenchymal transition
GLUTs: glucose transporters
HDAC: histone deacetylase
HOTAIR: HOX antisense intergenic RNA
IFN‐γ: interferon‐gamma
IL: interleukin
IRF: interferon regulatory factor
LINKA: long intergenic noncoding RNA for kinase activation
lncRNA: long noncoding RNA
lncRNA‐MIF: c‐Myc inhibitory factor
LPS: lipopolysaccharide
MALAT‐1: metastasis‐associated lung adenocarcinoma transcript 1
MDSCs: myeloid‐derived stem cells
MEG3: maternally expressed gene 3
NBR2: neighbour of BRCA1 gene 2
ncRNA: noncoding RNA
NeST: nettoie Salmonella pas Theiler's
NFAT: nuclear factor of activated T cells
NF‐κB: nuclear factor kappa‐light‐chain‐enhancer of activated B cells
NO: nitric oxide
NRON: non‐protein‐coding RNA, repressor of NFAT
NSCLC: non‐small‐cell lung carcinoma
PCGEM1: prostate cancer gene expression marker 1
PCR2: polycomb repressive complex 2
PVT1: plasmacytoma variant translocation 1
SOD2: superoxide dismutase 2
STAT3: signal transducer and activator of transcription 3
TBILA: TGF‐β‐induced lncRNA
TGF‐β: tumour growth factor‐beta
THRIL: TNF‐α‐ and hnRNPL‐related immunoregulatory lncRNA
Th: T helper
TLR: Toll‐like receptor
TME: tumour microenvironment
TNF‐α: tumour necrosis factor‐alpha
Tregs: regulatory T lymphocytes

## 1 Introduction

Non‐protein‐coding RNAs (ncRNAs) consist of multiple species that can be categorized into small noncoding RNAs and long noncoding RNAs (lncRNAs), based on a cut‐off at 200 nucleotides between the two categories. They are well known to possess regulatory functions, including splicing (small nuclear RNAs), nuclear organization (small nuclear and small nucleolar RNAs), transposon silencing (Piwi‐interacting RNAs) and inhibition of mRNA translation (microRNAs).

Long noncoding RNAs are the largest class of ncRNAs, with approximately 16 000 identified in humans and 9000 in mice, according to the latest release of the GENCODE project (www.gencodegenes.org). It remains unknown, however, how many of these transcripts have RNA‐mediated physiological effects (Elling *et al*., 2016). lncRNAs are also usually classified based on genomic location into seven broad but mutually nonexclusive categories (stand‐alone lncRNAs, natural antisense transcripts, pseudogenes, long intronic ncRNAs, divergent transcripts, promoter‐associated transcripts and enhancer RNAs) (Elling *et al*., 2016).

Long noncoding RNAs are sequences with low coding potential and conservation among species. Moreover, cumulative evidence has revealed important roles in post‐transcriptional gene modulation in several diseases. In particular, they appear to play major roles in cancer; multiple lncRNAs have been associated with various types of cancer. Alterations in lncRNA expression and their mutations promote tumorigenesis and metastasis. lncRNAs may be classified according to their structure or their mechanism of action and may exhibit tumour‐suppressive and tumour‐promoting (oncogenic) functions.

Recent studies also highlighted their role in immune cell differentiation and immune system function in cancer. For example, a growing number of papers have shown their roles in T cell functions during the progression of several solid tumours (including hepatocellular, lung and cervical cancers). Additionally, they play an important role in innate immunity by regulating natural killer (NK) cells (Zhang *et al*., 2016).

This review does not consider the genomic contexts of lncRNAs, neither does it list all lncRNAs involved in cancer immunity crosstalk. We aim to retrieve from the literature the best known lncRNAs that have specifically been associated with cancer immunity regulation, tumour metabolism and the tumour microenvironment (TME). Our selection aspires to show those markers that will be more likely to have a clinical impact in the near future and might be innovative targets for cancer therapy.

## 2 Materials and methods

A literature search was conducted using the Medline database, covering a period from January 2009 to July 2018. We chose 2009 as the starting point because, to the best of our knowledge, this was first time that a correlation among lncRNA and immunity was found.

The following Medical Subject Headings (MeSH) terms and keywords were used in the search: (‘T cells’ or ‘dendritic cells’ or ‘immune cycle’ or ‘immunity’) AND (‘long non‐coding RNA’ or ‘lncRNA’ or ‘ncRNA’).

We (a) considered only English as a language and we excluded from the analysis studies devoted to other pathologies, and (b) analysed those studies taking into account the previous reported reviews on this topic.

Electronic search results were supplemented with hand‐searching of selected papers, expert consensus meeting notes and reference lists from selected articles.

Data extraction was performed by the first author.

Data analyses were performed by each author through the compilation and discussion of the manuscript and its tables. All the authors wrote and approved the final manuscript.

## 3 Results

**Figure 1:**
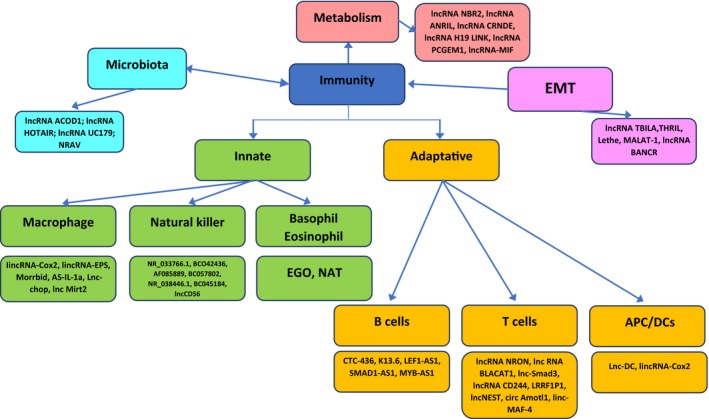
Role of most studied lncRNAs in cancer immunity Pink/violet/light blue boxes show reported interactions between microenvironment, lncRNAs and immunity. Green/yellow boxes show the interaction between innate and adaptive immunity and lncRNAs; they fight cancer cells directly through NK and T cell activation and indirectly through macrophages/B cells and DCs. lncRNAUC179, lncRNA transcribed‐ultraconserved region 179; NRAV: negative regulator of antiviral response; EGO: eosinophil granule ontogeny; NAT: natural antisense transcripts.

**Figure 2:**
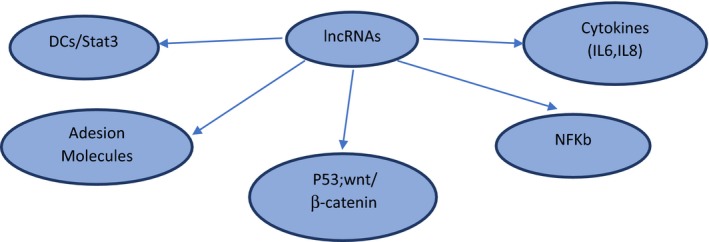
Examples of lncRNAs/cancer immunity interactions. lncRNAs are involved in transcriptional regulation of expression of cytokines (IL‐6, IL‐18, IL‐8, IL‐17)/adhesion molecules/IFN family/dendritic cell differentiation.

Long noncoding RNAs have a role in immunity both as a response to physiological and pathological stimuli (Table 1, Fig. 1 **)**. Many lncRNAs are only expressed in innate immune cells following their activation, while others, which are physiologically abundantly expressed, are downregulated when cells are exposed to inflammatory stimuli (Carpenter *et al*., 2013). Fig. 1 depicts the complex regulation by lncRNAs in cancer immunity. In Fig. 2, some key examples of lncRNAs/cancer immunity interactions are given.

**Table 1:** Major lncRNAs involved in immunity regulation. IQGAP, IQ‐motif‐containing GTPase‐activating protein 1; LRRK2, leucine‐rich repeat kinase 2; MBD1, methyl‐CpG‐binding domain protein 1; PACER, p50‐associated COX‐2 extragenic RNA; PTGS2, prostaglandin‐endoperoxide synthase 2; RasGEF1b, Ras‐GEF domain‐containing family member 1b; ICAM1, intercellular adhesion molecule 1; Bcl2l11, Bcl2‐like protein 11; hnRNPL/D, heterogenous nuclear ribonucleoprotein L/D

lncRNA	Type of cell	Function	Potential application	Reference
TBILA	Stromal cells	Upregulates TGF‐β; induces EMT in NSCLC	Target for anticancer therapies	Lu *et al*. (2018)
lnc‐ACOD1	Macrophages	Reduces viral load through IRF/IFN‐I‐independent pathway. It is involved in amino acid metabolism and tricarboxylic acid cycles	Target for anticancer and antiviral therapies	Li *et al*. (2016)
HOTAIR	Myeloid cells	Induces differentiation of granulocytes	Diagnostic marker	Zhang *et al*. (2014)
lnc‐CHOP	Myeloid cells	Induces immune suppression of MDSCs through activation of C/EBPβ and upregulation of arginase I, NO synthase, NADPH oxidase 2 and COX2	Target for anticancer therapies	Gao *et al*. (2018)
lnc‐DC	Dendritic cells	Regulates of DCs differentiation by activating STAT3	Target for anticancer therapies	Wang *et al*. (2014)
BLACAT1	T cells	Increases Treg infiltration activating the Wnt/β‐catenin pathway	Diagnostic marker	Su *et al*. (2017)
CD244	T cells	Regulates TNF‐α through modulation of chromatin methylation of the gene PCR2; inhibits expression of IFN‐γ	Diagnostic marker	Kang *et al*. (2017)
NeST	T cells	Regulates IFN‐γ accumulation and transcription by recruiting its promoter complex	Target for anticancer, antiviral and antibacterial therapies	Collier *et al*. (2012), Gomez *et al*. (2013)
Lethe	Stromal cells	Is induced by IL‐1β and TNF‐α and acts as a NF‐κB decoy molecule to limit inflammation. It binds nuclear RelA homodimers and prevents their accumulation at target gene loci, including IL‐6. It induces also transcription of IL‐8 and SOD2	Target for metabolic and anticancer therapies	Rapicavoli *et al*. (2013)
lnc‐COX2	Macrophages	Is induced downstream of TLR activation in macrophages and DCs to repress and activate large gene sets. It represses IL‐12β regulator chemokines (CCL5, CX3CL1), chemokine receptor (CCR1) and IFN‐γ‐stimulated genes (IRF7, Oas1α)	Diagnostic marker and target for anticancer therapies	Carpenter *et al*. (2013)
NRON	T cells	Restricts inappropriate activation of CD4+ T cells by sequestering phosphorylated NFAT in the cytoplasm in a large protein complex with IQGAP and LRRK2	Diagnostic marker	Willingham *et al*. (2005)
THRIL	Monocytes	Is activated after exposure to bacteria lipoteichoic acid; it regulates expression of inflammatory genes by recruiting hnRNPL	Target for anticancer therapies	Li *et al*. (2014b)
H19	Stem cells	Maintains long‐term stem cell quiescence and self‐renewal. H19 binds MBD1 and recruit methyltransferase complexes to place repressive methylation marks on target imprinted loci	Diagnostic marker and target for anticancer therapies	Monnier *et al*. (2013), Venkatraman *et al*. (2013)
PACER	Monocytes	Induces PTGS2 expression by sequestering NF‐κB p50 subunit	Target for anticancer therapies	Krawczyk and Emerson (2014)
RasGEF1b	Macrophages	Acts as a miRNA sponge that targets ICAM1 to regulate expression of inflammatory molecules	Target against microbial infection and for anticancer therapies	Ng *et al*. (2016)
Morrbid	Myeloid cells	Regulates lifespan of neutrophils, eosinophils, monocytes by repressing Bcl2 by promoting the enrichment of the PRC2 complex at the Bcl2l11 promoter to maintain this gene in a poised state.	Target for anticancer and inflammatory syndrome therapies	Kotzin *et al*. (2016)
lnc13	Macrophages	Binds to hnRNPD to suppress transcription of immune response genes	Diagnostic marker	Castellanos‐Rubio *et al*. (2016)

The importance of this paper lies in its aim, as it attempts to systematically correlate the change in lncRNA expression with different aspects of the immune system and cancer immune response. We will also underline functions and/or specific lncRNAs whose expression is implicated in cell migration, invasion and epithelial–mesenchymal transition (EMT) processes and that might represent novel targets of antitumoural therapy.

### 3.1 Role in the tumour microenvironment

In the TME, lncRNAs regulate tumour necrosis factor‐alpha (TNF‐α) and interact with the NF‐κB pathway, mainly in a Toll‐like receptor (TLR)‐dependent manner. TLRs induce the expression of numerous lncRNAs, such as lincRNA‐Cox2 (Johannessen *et al*., 2013).

In order to identify and characterize lncRNAs whose expression is changed following the induction of the innate immune response, Roux *et al*. (2017) evaluated, in humans and in mice, the lncRNA profiles of different cells after lipopolysaccharide (LPS) and IL‐1 exposure (Roux *et al*., 2017). These authors performed a huge sequencing analysis obtaining 204 lncRNAs that were differentially expressed across four human cell types (monocytes, macrophages and two stromal cell types). They were able to identify 111 novel lncRNAs. The majority (161 lncRNAs) were expressed in a cell‐specific manner, but 43 lncRNAs were induced in multiple cell types. Positional analysis revealed that lncRNA expression correlated with immune‐related genes, therefore suggesting that these might be functionally linked (Roux *et al*., 2017).

### 3.2 Role in epithelial–mesenchymal transition

An increasing number of lncRNAs being explored are implicated in EMT regulation. EMT occurs in neoplastic cells that have previously undergone genetic and epigenetic changes; it leads to enhanced migratory capacity, invasiveness and elevated resistance to apoptosis and greatly contributes to an immunosuppressive microenvironment.

Initial reports indicated lncRNA involvement in the transforming growth factor‐beta (TGF‐β) signal pathway. However, the critical pathways of EMT and tumour cell metastasis remain poorly understood.

Major studies showed that lncRNA LINC01186 and lncRNA‐HIT (HOXA transcript induced by TGF‐β) are mediators of TGF‐β signalling in lung and breast cancer cells, respectively, playing a significant role in the regulation of lung and breast cancer cell migration and invasion (Hao *et al*., 2017; Richards *et al*., 2015).

It seems that lncRNAs may act through competing endogenous RNAs (ceRNAs) for miRNAs or through mediating epigenetic silencing by recruiting the polycomb repressive complex 2 (PRC2) (Heery *et al*., 2017; Xu and Cao, 2018). Several lncRNAs have now been found to interact with PRC2 by recruiting enhancer of zeste homolog 2 (EZH2) or other components, and this mechanism is also a major way by which lncRNAs regulate EMT (Battistelli *et al*., 2017; Li *et al*., 2017a,b; Wang *et al*., 2015a; Yang *et al*., 2018).

In a Chinese study presented at the last American Society for Clinical Oncology Congress, a comparison of global gene expression patterns of non‐small‐cell lung carcinoma (NSCLC) cells treated with and without TGF‐β demonstrated 423 lncRNAs to be upregulated and 246 downregulated in TGF‐β‐treated cells compared with control cells. One of the most prominent hits, named TGF‐β‐induced lncRNA (TBILA), promoted NSCLC progression and was upregulated in tumour tissues (*P* < 0.001). TBILA is upregulated by the TGF‐β classical signalling pathway and promotes human germinal centre‐associated lymphoma expression by binding with the Smad transcription factor complex, thereby enhancing RhoA activation (Lu *et al*., 2018).

Many bioinformatic studies have confirmed the disease‐specific roles of different lncRNAs in tumorigenicity and metastasis *in vitro* and *in vivo*. For example, in basal‐like breast cancer, specific lncRNAs correlate with the activation of epidermal growth factor receptor‐dependent pathways and EMT (Ouyang *et al*., 2018).

### 3.3 Role in metabolism

Cancer immunity and metabolic pathways mutually influence one another. Metabolites do not only provide energy and substrates for growth and survival, but also instruct effector immune functions, differentiation and gene expression. Mounting evidence shows that lncRNAs can regulate glucose metabolism in cancer cells in different ways, such as by directly regulating glycolytic enzymes (i.e. pyruvate carboxylase, fructose‐2,6‐bisphosphatase, 6‐phosphogluconate, phosphoenolpyruvate carboxykinase) and glucose transporters (GLUTs), or by indirectly modulating the signalling pathways (Wnt/Snail, STAT and p53 pathways and HIF, PI3K/AKT/mTOR and LKB1‐AMPK pathways) (Fan *et al*., 2017).

Accumulating evidence indicates that lncRNAs also regulate glucose metabolism in tumour cells, with particular regard to the Warburg effect (Hua *et al*., 2018). The metabolic remodelling in a tumour niche is endured not only by cancer cells but also by noncancerous cells that share the same microenvironment; in particular, macrophages seem to increase aerobic glycolysis via AKT/mTOR and HIF‐1α stabilization. Moreover, immune cells in the microenvironment enhance fatty acid biosynthesis and uptake and increase the expression of genes involved in glutamate transport and metabolism. It seems that immune cells might also induce the overexpression of arginase 1 (ARG1) (Wenes *et al*., 2016).

Although there is adequate oxygen, cancer cells often generate energy through glycolysis, rather than oxidative phosphorylation. Glycolysis minimizes reactive oxygen species production in mitochondria and promotes a more acidic extracellular pH via lactic acid release, establishing an appropriate TME for tumour growth, invasion and metastasis. This process also impairs T cell function (Yu *et al*., 2017). Moreover, EMT transcriptional factors may exacerbate glucose metabolism dysregulation by targeting glycolytic enzymes. For example, Twist promotes glucose metabolism reprogramming by activating the β1‐integrin/FAK/PI3K/AKT/mTOR pathway and inhibiting p53 signalling (Yang *et al*., 2015), while Snail can serve as a positive regulator of fructose‐1,6‐bisphosphatase (FBP1) and is critical for E‐cadherin promoter silencing (Dong *et al*., 2013).

In breast cancer, lncRNA NBR2 (neighbour of BRCA1 gene 2) overexpression upregulates GLUT1 expression through stimulating AMPK activity (Fan *et al*., 2017). Upregulation of GLUT1 expression via PI3K/AKT/mTOR signalling may also depend on lncRNA ANRIL (antisense noncoding RNA in the INK4 locus), resulting in greater glucose uptake and utilization (Zou *et al*., 2016). lncRNA CRNDE (colorectal neoplasia differentially expressed) positively modulates GLUT4 expression via epigenetic modifications, thereby promoting metabolic changes (Ellis *et al*., 2014). According to recent studies, glucose transporter and certain enzymes involved in aerobic glycolysis are regulatory targets of lncRNAs; these enzymes include hexokinase 2 (HK2), lactate dehydrogenase A, pyruvate kinase isoenzyme M2 (PKM2) and pyruvate dehydrogenase kinase 1 (PDK1). The Warburg effect is also promoted by lncRNA PVT1 (plasmacytoma variant translocation 1); lncRNA H19 (Li *et al*., 2014a); LINKA (long intergenic noncoding RNA for kinase activation) (Lin *et al*., 2016); lncRNA PCGEM1 (prostate cancer gene expression marker 1) (Hung *et al*., 2014); and lncRNA‐MIF (c‐Myc inhibitory factor) (Zhang *et al*., 2016).

Many lncRNAs can directly or indirectly regulate p53 expression: for example, lncRNA MEG3 (maternally expressed gene 3), which can increase P53 protein (Zhang *et al*., 2010); and lincRNA‐p21, MALAT‐1, ROR and CASC9, which have also been found to regulate glycolysis via HIF‐1α or p53.

Thus, despite the emerging role of lncRNAs in the regulation of glucose and lipid metabolism, as well as hormonal and nutritional signalling pathways, their significance in metabolism biology awaits further confirmation. Understanding the role of lncRNAs in regulating cancer metabolism is critical to explore the possibility of using the anti‐Warburg effect of certain lncRNAs for targeted therapy, as they may slow down the cell cycle by blocking the G1/S and G2/M transitions.

### 3.4 Role in microbiota

Host/microbiota/cancer interaction is an area of emerging interest. An unsuspected link between cancer and microbiota has been described in the last decade. Microbes and the microbiota may amplify or mitigate carcinogenesis, and may also influence the responsiveness to cancer therapeutics and/or affect cancer‐associated complications.

Long noncoding RNAs play a fundamental role in microbiota equilibrium: they are prominently involved in the response of different host cells to various bacterial agents. However, how they interact with the immune response is unclear. Several lncRNAs, as well as some TLR ligands, have recently been implicated in the implied innate responses to various bacteria such as *Mycobacterium* (M.) spp., *Salmonella* (S.) *Typhimurium*,* Escherichia coli*,* Listeria* (L.) *monocytogenes*,* Helicobacter pylori*, and *Campylobacter* (C.) *concisus*; in addition, several studies have demonstrated the involvement of TLR ligands, but their function remains to be elucidated (IIott *et al*., 2014; Westermann *et al*., 2016; Yang *et al*., 2016; Yi *et al*., 2014; Zhu *et al*., 2015; Zur Bruegge *et al*., 2017). For example, 221 out of 989 lncRNAs showed LPS‐induced differences in expression levels (IIott *et al*., 2014).

No study has yet compared lncRNA expression in response to different pathogens possessing different virulence mechanisms. During infection, a complex interplay of microRNA and lncRNAs occurs: for example, miR‐155 and miR‐146 are induced in a NF‐κB‐dependent pathway promoting or dampening inflammation (Duval *et al*., 2017).

Liang *et al*. (2015) showed correlation among lncRNA expression and gut microbes, demonstrating the ability of cellular machinery to distinguish between different microbes. They compared type‐specific expression patterns of lncRNAs between reconventionalized mice and gnotobiotic mice. They found only six lncRNAs commonly upregulated in both types of mice, although 613 lncRNAs were upregulated in at least one condition. The authors observed that these six lncRNAs were highly expressed in immune organs (such as the spleen and thymus), leading them to suggest that these lncRNAs might be involved in host immune responses. It was supposed that lncRNA signatures in response to gut microbes arise from host–microbe interactions (Liang *et al*., 2015).

Wang *et al*. (2017) showed that lncRNA aconitate decarboxylase 1 (ACOD1) might be induced by viral infection through a pathway that is independent of interferon regulatory factor 3 (IRF3)/type I IFN (IFN‐I) signalling, but dependent on the NF‐κB‐dependent pathway. The authors reported that *in vitro* or *in vivo* deficiency in lncRNA ACOD1 significantly reduced viral load in macrophages and in immune organs through an IRF3/IFN‐I‐independent pathway, and the lncRNA ACOD1 directly interacts with glutamic‐oxaloacetic transaminase 2 (GOT2), which is involved in amino acid metabolism and tricarboxylic acid (TCA) cycles during viral infection (Wang *et al*., 2017).

Additionally, it was suggested that HCV core protein might induce lncRNA HOX antisense intergenic RNA (HOTAIR), which is followed by a decrease in expression of Silent information regulator 1 (Sirt1), a histone deacetylase (HDAC) that modulates glucose‐ and lipid‐metabolism‐related gene profiles, resulting in metabolic disorders in hepatocytes (Li *et al*., 2016).

### 3.5 Role in dendritic cell and myeloid‐derived stem cell regulation

Dendritic cells (DCs) are responsible for initiating a number of antigen‐specific immune responses. They work as regulators of antigen presentation and are also adept at generating the appropriate amount of T cells in response to a given pathogen. Therefore, they play a critical role in immune balance between pathogen elimination or escape.

In the last few years, several papers have shown that multiple lncRNAs may regulate DCs functions and immune tolerance; these lncRNAs include lnc‐DC, lincRNA‐Cox2, lincRNA‐EPS and AS‐IL‐1α in macrophages, and lncRNA Morrbid in myeloid‐derived stem cells (MDSCs) (Atianand *et al*., 2016; Carpenter *et al*., 2013; Chan *et al*., 2015; Kotzin *et al*., 2016; Wang *et al*., 2014).

Carpenter *et al*. (2013) showed that lincRNA‐Cox2 regulates more than 700 genes, including chemokines (CCL5, CX3CL1), chemokine receptors (CCR1) and interferon‐stimulated genes (ISGs) (IRF7, Oas1α, Oas1l, Oas2, Ifi204 and Isg15), in macrophage cell lines. All of these genes were observed to be upregulated when lincRNA‐Cox2 was silenced in unstimulated cells (Carpenter *et al*., 2013).

The differentiation of granulocytes has been reported to be partly mediated by HOX antisense intergenic RNA myeloid 1 (HOTAIRM1), an antisense lncRNA within the HOXA gene locus. HOTAIRM1 knockdown abrogated retinoic acid‐dependent activation of HOXA1/A2 and CD11b and CD18 (Mac‐1), which are two β2‐integrin transcripts that are associated with myeloid maturation (Tian *et al*., 2018; Zhang *et al*., 2009).

Gao *et al*. (2018) identified a novel lncRNA, named lnc‐CHOP, in MDSCs; lnc‐CHOP has an important role in controlling the immunosuppressive function of MDSCs in the tumour environment. lnc‐CHOP binds with both CHOP and the C/EBPβ isoform liver‐enriched inhibitory protein, thereby promoting the activation of C/EBPβ and upregulating the expression of ARG1, nitric oxide (NO) synthase 2, NADPH oxidase 2 and cyclooxygenase‐2 (COX2), which are related to the immunosuppressive function of MDSCs in inflammatory and tumour environments (Gao *et al*., 2018).

Wang *et al*. (2014) studied the role of lnc‐DC in the mouse. Wang and colleagues reported that knockdown of lnc‐DC impaired DCs differentiation from human monocytes *in vitro* and from mouse bone marrow cells *in vivo*, and also reduced the capacity of DCs to stimulate T cell activation. These effects were mediated via lnc‐DC‐induced activation of the transcription factor STAT3 (signal transducer and activator of transcription 3). lnc‐DC binds directly to STAT3 in the cytoplasm, thereby preventing the latter from binding to and being dephosphorylated by SHP1, and thus promoting phosphorylation of STAT3 on tyrosine‐705. As STAT3 is one of the most important pathways of the immune‐suppressive TME, this lnc‐DC deserves further study (Wang *et al*., 2014).

### 3.6 Role in T and B cell regulation

T cell activity is of crucial importance in the TME and correlates with outcomes in almost all tumours. The ratio between effector and regulatory T cells (Teff/Treg) correlates with survival in both solid and haematologic tumours.

Recent findings support the importance of lncRNAs as mediators of several T cell functions. Some lncRNAs may affect T cell‐induced cell death and impair cytotoxic functions, or regulate Treg differentiation. Hu *et al*. (2013) identified 1524 lncRNAs in 42 T cell samples ranging from early T cell progenitors to terminally differentiated T helper (Th) subsets. STAT4 was found to activate the expression of Th1‐preferred lncRNAs, while STAT6 activated the expression of Th2‐preferred lncRNAs (Hu *et al*., 2013). Th2‐locus control region lncRNAs establish histone H3K4Me marks at the IL‐4, IL‐5 and IL‐13 promoters and recruit enzymes for alternatively splicing to enable Th2 cells to express IL‐4, IL‐5 and IL‐13 (Hu *et al*., 2013).

Long noncoding RNA NRON negatively regulates T cell activation by interacting with the nuclear factor of activated T cells (NFAT), which is localized to the cytoplasm and imported into the nucleus in response to calcium‐dependent signalling. NRON inhibits nuclear accumulation of NFAT by either binding to nuclear transport factors or sequestering inactive NFAT in the cytosol (Willingham *et al*., 2005). In several cancers, lncRNAs increase Tregs through the activation of the Wnt/β‐catenin signalling pathway. For example, in cervical cancer, the lncRNA BLACAT1 promotes proliferation, migration and invasion by modulating this pathway (Wang *et al*., 2018).

In Treg cells, lnc‐Smad3 interacts with the histone deacetylase HDAC1 and contributes to epigenetic modifications, thereby silencing the expression of Smad3, which can mediate signals from the TGF‐β superfamily ligands to regulate cell activity (Xia *et al*., 2017).

lncRNA‐CD244 induces T cell‐inhibitory signalling in tuberculosis infection. It regulates TNF‐α through the modulation of chromatin methylation states of the subunit EZH2 of PCR2 (Wang *et al*., 2015b). Shi *et al*. (2014) showed that an unnamed lncRNA is regulated through binding with LRRFIP1 (a repressor of TNF‐α) and chromatin.

Long noncoding RNAs may have a scaffold role with EZH2, SUZ12 and LRRFIP1, mediated by assembling on an RNA tether to the region; for example, the lncRNA NeST has been shown to switch chromatin to an active state by binding WD repeat‐containing protein 5 (WDR5) in the histone 3 lysine 4 methyltransferase complex (H3K4), and this leads to IFN‐γ accumulation. NeST negatively regulates T cell cytotoxic function (Gomez *et al*., 2013).

Transcriptional repression of target genes is dependent on interactions of lincRNA‐Cox2 (located upstream of the Cox2 coding gene) with heterogeneous nuclear ribonucleoprotein A/B and A2/B1.

Aberrant lncRNA expression was documented in B cells (Isin *et al*., 2014; Tayari *et al*., 2016). lncRNAs also regulate gene expression in B cells, although B cell regulators differ from those involved in T cell regulation. Brazao *et al*. (2016) showed that 20% of lncRNAs identified in B cells are associated with enhancer or promoter regions to regulate gene transcription (Brazao *et al*., 2016).

Unlike mRNA expression patterns, lncRNA expression patterns can distinguish between cells committed to the B and T cell lineage as early as the progenitor stage in bone marrow (Casero *et al*., 2015), indicative of their importance in lineage commitment. During later stages of B cell development and maturation, lncRNA expression profiles can be very similar between functionally distinct B cells such as follicular and marginal zone B cells in the spleen (Brazao *et al*., 2016) and naïve and memory cells in tonsils (Tayari *et al*., 2016).

Petri *et al*. (2015) identified different lncRNAs at different B cell development stages: lncRNAs CTC‐436K13.6, LEF1‐AS1, SMAD1‐AS1 and MYB‐AS1 were associated with preBI, preBII and immature cells, while lncRNAs OIP5‐AS, MME‐AS1 and the bidirectional lncRNA CRNDE were associated with more advanced stages of development (Petri *et al*., 2015).

### 3.7 lncRNAs and the NF‐κB pathway

NF‐κB (nuclear factor kappa‐light‐chain‐enhancer of activated B cells) is a protein complex that controls cytokine production, DNA transcription and cell survival. It plays a key role in regulating the immune response to infection and is involved in cellular responses to chemical, physical and microbiological stimuli. NF‐κB is also an actor in lncRNA networks. lncRNAs interfere with pathways that involve NF‐κB function both directly (e.g. NKILA) and indirectly, through prostate transmembrane protein, androgen induced 1 (PMEPA1) regulation (Liu *et al*., 2015).

Guttman *et al*. (2009) reported a direct association between lincRNA‐Cox2 and TLR4 stimulation via NF‐κB (Guttman *et al*., 2009). lincRNA‐Cox2 mostly regulates genes in macrophages exposed to LPS or TLR2 (Tong *et al*., 2016). Much like observations in macrophages, knockdown of lincRNA‐Cox2 results in reprogramming of gene expression profiles in intestinal epithelial cells exposed to TNF‐α. lincRNA‐Cox2 appears to repress IL‐12β transcription; lincRNA‐Cox2 mediates these effects via its interactions with the Mi‐2/nucleosome remodelling and deacetylase (Mi‐2/NuRD) repressor complex; LincRNA‐Cox2 seems to guide the complex to the IL‐12β promoter region (Tong *et al*., 2016).

Much like lincRNA‐Cox2, THRIL (TNF‐α‐ and hnRNPL‐related immunoregulatory lncRNA) is another inducible lncRNA that acts in part via its interaction with hnRNPL. This lncRNA was identified in the THP‐1 human monocyte cell line and is one of 159 lncRNAs that are differentially expressed upon activation with Pam3CSK4 (bacterial lipoteichoic acid or lipoprotein) treatment (Li *et al*., 2014b).

Several distinct lncRNAs have been reported to modulate the levels of the IL‐1 family of pro‐inflammatory cytokines (Chan *et al*., 2015). The lncRNA Mirt2 functions as a checkpoint to prevent aberrant activation of inflammation and is a potential regulator of macrophage polarization. lncRNA AS1 enhances IL‐1α gene transcription, and disruption of AS‐IL‐1α function can limit IL‐1α transcription and potentially alleviate the damaging effects of excessive IL‐1α levels during infection and inflammatory disease (Chan *et al*., 2015; Du *et al*., 2017). A recently identified lncRNA, named Lethe, binds the NF‐κB subunit p65 (RelA) preventing it from directing transcription of IL‐6, IL‐8 and SOD2 (Rapicavoli *et al*., 2013).

The question of whether lncRNA interference with NF‐κB and other immune‐related pathways favours the host or the pathogen needs to be further investigated. The lncRNAs expressed upon TLR stimulation may act to fine‐tune the immune function, as previously described for microRNAs (Siddle *et al*., 2015).

### 3.8 circRNAs

Circular RNAs (circRNAs) are a novel class of endogenous noncoding RNAs, characterized by their covalently closed loop structures without a 5′ cap or a 3′ poly(A) tail, formed through self‐ligation of the 3′ and 5′ ends, as generated through back splicing.

Potential roles for circRNAs in regulating genes encoding inflammatory molecules have also been reported. It was previously shown that delivery of purified circRNA stimulates a greater innate immune response than that stimulated by linear RNA with the same sequence. In addition, circRNA produced in the cell by foreign introns induces the expression of immune genes. circRNAs were shown to soak up and suppress some miRNAs and RNA‐binding proteins (Ashwal‐Fluss *et al*., 2014). Finally, circRNAs were thought to be more prone to adopt secondary or tertiary structures due to the constrained circular geometry, so the same circRNA generated by endogenous introns is recognized as ‘self’ and is associated with a set of diverse RNA‐binding proteins. Moreover, Chen *et al*. (2017) evidenced an unsuspected antiviral property. Intriguingly, circRNAs made with self‐splicing introns stimulated the innate immune response through RIG‐I (retinoid‐induced genes) (Chen *et al*., 2017). These results were confirmed by Li *et al*. (2017a,b), who found circRNA biogenesis to be affected by both nuclear NF90/110 and non‐nuclear proteins, such as RIG‐I and TLR3 (Li *et al*., 2017a). One study catalogued circRNA expression in macrophages, identifying nearly 2000 circRNAs induced by TLR4 stimulation (Ng *et al*., 2016). circAmotl1 is a circRNA involved in STAT3 signalling; it increases STAT3 expression and stimulates its nuclear translocation to regulate the expression of mitosis‐associated genes. It was suggested that circAmotl1 may promote nuclear c‐myc expression in cancer cell lines, thereby helping this factor bind to gene promoters to trigger tumorigenicity (Yang *et al*., 2017).

## 4 Discussion

Immune system regulation is complex; antimicrobial and anticancer defences require rapid changes in gene expression in order to modify the microenvironment component. The production of cytokines, chemokines and additional immune mediators leads to an inflammatory environment, in which metabolic pathways are also activated. After activation, the immune system turns off to avoid immune‐mediated damage to the host. An imbalance of immune status in the TME contributes to the development and progression of cancer, and this is the basis of many studies of immunotherapy.

Long noncoding RNAs have been shown to be potential key regulators in the processes of proliferation, migration and invasion of cancer cells by regulating gene expression patterns at various levels, including chromatin‐organizational, transcriptional and post‐transcriptional regulation (Denaro *et al*., 2014). There are multiple lines of evidence that lncRNAs can function as oncogenes (e.g. HOTAIR, MALAT‐1/NEAT, H19, PVT1) or tumour suppressor genes (e.g. MEG3, GAS5, lincRNA‐p21, PTENP1) (Beermann *et al*., 2016; Jiang *et al*., 2017). Moreover, the expression of some lncRNAs correlates with poor prognosis and resistance to treatment; this behaviour probably reflects an immune‐resistant microenvironment. Schmitt and Chang (2016) divided lncRNAs into (a) those associated with cancer genomic alterations, (b) predictors of therapeutic responsiveness, (c) prognosticators and (d) markers of diagnosis and monitoring (Schmitt and Chang, 2016).

Moreover, lncRNAs act as regulators of cancer immunity processes. In addition to their regulation in cancer development and metastasis, recent studies have suggested that lncRNAs play crucial roles in different phases of cancer immunity, including (a) infiltration into cancer tissues, (b) antigen presentation, (c) antigen release, (d) immune activation, (e) immune cell migration and (f) killing of cancer cells.

Analysis of the literature indicates that lncRNAs occupy a central role in innate and adaptive immunity regulation, as well as in the development, progression and maintenance of many human tumours (Yu *et al*., 2018). Reciprocal crosstalk between the immune system, EMT and metabolism regulation synergistically contributes to malignant cancer behaviours, but the regulatory mechanisms underlying this interaction remain unclear. While many studies have provided new insights into immune gene regulation by lncRNAs, the vast majority of lncRNAs in the immune system remain largely uncharacterized. We might conclude that lncRNAs are a novel but fundamental part of cancer immunotherapy, and their tissue‐ and target‐specific nature enhances their utility as both diagnostic markers and therapeutic agents.

## 5 Conclusion

Our understanding of how lncRNAs influence the immune system is very recent, and it remains unknown whether there are specific lncRNAs expressed in response to a certain pathogen or whether lncRNAs are mainly involved in basic cellular immune responses to different stress stimuli.

Recent evidence showed that lncRNAs can regulate inflammation and innate immunity by targeting various metabolic pathways in different manners, either functioning through cis‐regulation (e.g. βlinc), antisense inhibition (e.g. AdipoQ AS and IDH1‐AS), interaction with proteins (e.g. lncRNA‐ACOD1, SAMMSON and NBR243) or interaction with miRNA sponges (e.g. H19).

It has been shown that lncRNAs regulate TNF‐α and interact with the NF‐κB pathway mainly in a TLR‐dependent manner in the TME. Moreover, they regulate EMT via the TGF‐β signalling pathway. lncRNAs also control glucose metabolism in tumour cells and are involved in the response of different host cells to various bacterial agents. Their role in the regulation of cytotoxic T cells and Tregs is fundamental, but they also affect B cell functions. The regulation of macrophage activity involves chemokines and IFN‐stimulated genes. The functional importance of immune‐related lncRNAs is just beginning to be characterized; their role as both biomarker and target in the immunotherapy of cancer needs to be examined further. Future studies are warranted to deepen our understanding of lncRNA immune functions and their application in clinics.

## Author contributions

ND extracted data from public literature databases; ND, MCM and CLN contributed to data analyses and discussion; ND and CLN prepared the manuscript; ND, MCM and CLN: approved the final manuscript.

## Conflicts of interest

The authors have no conflicts of interest to declare.
